# Internal carotid artery dissection following chiropractic treatment in a pregnant woman with Systemic Lupus Erythematosus

**DOI:** 10.1186/2045-709X-20-38

**Published:** 2012-12-19

**Authors:** Adam Morton

**Affiliations:** 1 QLD Diabetes Centre Mater Health Services Raymond Terrace, South Brisbane, QLD, 4101, Australia

**Keywords:** Carotid dissection, Neck manipulation, SLE, Pregnancy

## Abstract

A case of internal carotid artery dissection in a pregnant woman with Systemic Lupus Erythematosus (SLE) immediately following chiropractic treatment is presented. The literature regarding complications of neck manipulation during pregnancy, spontaneous dissection of craniocervical arteries in pregnancy and the postpartum period, and dissection of craniocervical arteries in SLE are reviewed. To the best of the author’s knowledge, this is the first case of carotid artery dissection following chiropractic treatment in a pregnant woman published in the literature.

## Background

Dissection of the internal carotid artery accounts for approximately 20% of cases of ischaemic stroke in young adults. A population-based study reported the average annual incidence rate for spontaneous internal carotid artery dissection was 1.72 per 100 000 individuals [1]. Several vascular and connective tissue disorders have been associated with dissection, in particular migraine, fibromuscular hyperplasia and vascular Ehlers-Danlos syndrome. In addition, a number of physical activities have been associated with dissection. It remains unclear as to whether chiropractic neck manipulation is associated with craniocervical artery dissection. This paper describes a case of internal carotid artery dissection following chiropractic treatment in a pregnant woman with Systemic Lupus Erythematosus. The possible contributing factors in this case are discussed.

## Case presentation

A thirty-one year old woman, pregnant at 16 weeks gestation, presented to a chiropractor for treatment of occipital headache. She had suffered with intermittent bilateral occipital muscle tension headaches occurring monthly since the age of 14 years. The frequency of headache was unchanged during pregnancy. In addition she had a history of migraine characterised by unilateral frontal headache, the last episode of which had been 6 weeks earlier. There was no recent history of viral illness and no family history of stroke. The subjects history was also significant for Systemic Lupus Erythematosus (SLE) diagnosed twelve years earlier, complicated by renal involvement treated with azathioprine and prednisone, hypertension managed with labetalol, and episodes of deep vein thrombosis and pulmonary embolism. She was heterozygous for prothrombin gene mutation but did not have a lupus anticoagulant or anticardiolipin antibody.

Immediately after receiving treatment the subject noted severe right sided anterior neck pain, and rapidly developed ipsilateral Horner’s syndrome. It was not possible to obtain exact information regarding the nature of the chiropractic treatment; though from the subject’s description it is likely that spinal manipulation was given. Magnetic resonance imaging (MRI) revealed extensive dissection of the right internal carotid artery, extending from 5 cm distal to the carotid bulb to the horizontal intrapetrous segment. The subject was admitted to the intensive care unit and treated with intravenous heparin and subsequently low-molecular weight heparin. A flare in SLE was evidenced by a rise in blood pressure, a slight deterioration in maternal renal function, increasing proteinuria and a fall in platelet count. Four days after the onset of neurological symptoms intrauterine foetal demise was noted. The patient was subsequently anticoagulated with warfarin for six months. Follow-up MRI six months later revealed a focal false aneurysm of the right internal carotid artery. The Horner’s syndrome persists one year after the initial presentation.

## Discussion

Only one case of antepartum internal carotid artery dissection has been reported of which the author is aware [2]. Maderia et al described a 38 year old woman in her fifth pregnancy at 21 weeks gestation who presented with headache and a spontaneous left internal carotid artery dissection involving the petrous and cavernous segments on MRI. In addition 22 cases of postpartum carotid artery dissection have been reported [3,4]. There was an even distribution of vaginal and caesarean deliveries, the time from delivery to onset of symptoms ranged from 2 – 21 days. In eight cases there were additional possible contributing factors to dissection, these being reversible cerebral vasoconstriction syndrome (2 cases), posterior reversible encephalopathy syndrome (2), pre-eclampsia, infection, Ehlers-Danhlos syndrome and rheumatoid arthritis.

Possible predisposing factors to arterial dissection in the peripartum period include intimal injury related to the Valsalva manoeuver during labour, alterations in arterial wall integrity due to pregnancy-related hormonal or vasoactive substances, increase in cardiac output and blood volume and the hypercoaguable state of pregnancy [4].

Three cases of craniocervical arterial dissection have been reported in the setting of SLE or antiphospholipid syndrome, although one of these patients also had Takayasu’s arteritis. In addition four cases of spontaneous coronary artery dissection and twenty-one cases of aortic dissection have been described in individuals with SLE. Hypertension, glucocorticoid use and dyslipidaemia may be factors in individuals with SLE that may lead to atherosclerosis weakening the arterial wall. [5]. Vasculitis leading to chronic inflammation increasing vessel wall fragility has also been proposed as a contributing factor [6].

Migraine was shown to be associated with a two-fold increase risk of cervical artery dissection in a recent meta-analysis [7].

Stuber et al recently published an critical review of the literature regarding adverse effects from spinal manipulation in the pregnant and postpartum periods [8]. They identified adverse events in five pregnant women, and two postpartum women. The four serious events reported in the literature all occurred after cervical spine manipulation. These included cerebellar infarction, vertebral artery occlusion, odontoid fracture and a cervical epidural haematoma. They concluded that significant life-threatening injuries were rare following spinal manipulation during pregnancy and the postpartum period, and that further research into the frequency of adverse events and efficacy of spinal manipulation therapy in this population is necessary.

The controversy regarding the possible association between cervical spine manipulation and neurovascular complications remains unresolved and continues to be debated [9,10]. Conclusive evidence is difficult to obtain because of the rarity of adverse events. A population-based case-control and case-crossover study found no evidence of excess risk of vertebrobasilar stroke associated with chiropractic care compared with primary care physician visits [11].A recent retrospective case-control study, however, found an odds ratio of 12.8 (p = 0.009) for neck manual therapy in individuals less than 55 years of age presenting with craniocervical arterial dissection [12].

## Conclusions

In conclusion, the first case of internal carotid artery dissection following chiropractic treatment in a pregnant woman with SLE is described. Causality relating the chiropractic treatment to the craniocervical dissection cannot be established. The mother’s underlying medical condition, her immunosuppressive treatment with prednisone and the hormonal changes of pregnancy may all have contributed to vessel fragility, and her risk of cervical artery dissection was increased in view of her history of migraine. It is possible that the arterial dissection had occurred prior to seeking treatment, and may have become evident if the patient had sought the care of a general practitioner rather than a chiropractor. Practitioners should be aware of this possible complication of neck manipulation in pregnancy and the postpartum period, particularly in mothers with underlying medical disorders that may predispose to vessel fragility and arterial dissection.

### Consent

Written informed consent was obtained from the patient for publication of this Case report. A copy of the written consent is available for review by the Editor-in-Chief of this journal.

## Competing interests

The author declares that he has no competing interests.

## Authors’ contributions

AM cared for the patient, performed the literature review, and prepared the manuscript.
